# New Methods of Mounting Artificial Teeth

**Published:** 1880-04

**Authors:** 


THE
AMERICAN JOURNAL
OF
DENTAL SCIENCE.

Vol. XIII. THIRD SERIES—APRIL, 1880. No. 12.

### ARTICLE I.

### *New Methods of Mounting Artificial Teeth.*

[Transactions of Ohio State Dental Society.]

Dr. J. Taft.—I desire to make an inquiry in reference to this subject. Some months ago Dr. Holbrook, of Wisconsin, came to Cincinnati with what he claimed a superior mode of preparing plates, more especially in regard to their attachment to the mouth. Some of the members present have made experiments with this process. Dr. Berry and some others, perhaps have done so. In behalf of his process he had some very strong statements from men high in the profession whose opinions I have not been disposed to overlook or disregard. For instance, he had a very commendatory statement from Dr. Haskell, of Chicago. Now those who know Dr. Haskell would not suspect him of trumping up a statement in reference to a matter of that kind. Dr. Haskell makes the insertion of teeth on artificial plates a specialty almost, and continuous gum-work is the material he employs more than any other, and he claims that the method which was suggested is very good.

The method consists simply in trimming either the impression or the model at certain points to relieve the bearing and perhaps to some extent, to make space—at least to relieve the plate everywhere in the mouth where there would be undue pressure upon the hard parts. For lower plates it was considered especially good, as with it we can in almost all cases, not only have the plate set well in the mouth, but give adhesion by atmospheric pressure, same as with the upper plates. I did not see experiments enough to have much knowledge of it. He made experiments with one or two extreme cases at our office, but did not get the success he expected, notwithstanding at the outset he requested to have what were regarded as the very worst cases. We gave him a couple, not of the worst sort, but not by any means good cases, and the plates, though worn still, I believe in both cases, do not set much better than those used before. I desire to know if anybody knows anything experimentally about the utility of this method. We have had two or three other statements from other members of the profession in reference to it.

Another method is advertised by some man in Chicago, in which he claims to be able to insert in the mouth artificial sets of teeth without the large plates ordinarily used, and firmly, so that they will be serviceable. I do not remember his name: If anybody here does, I would like to hear something about it.

Dr. L. Buffett.—What Dr. Taft has stated is nothing new to me. I commenced about twenty-two years ago to study dentistry, and the doctor I was with would take a model in his hand and examine the mouth, and wherever he saw it would fit closely on the hard tissues he would trim the soft portions and also instructed his pupils to do the same. I think it is nothing more than Dr. Taft used to do.

Dr. W. M. Herriott.—Did you mean the impression or the model?

Dr. Buffett.—I meant the model.

Dr. Berry.—I believe in re-inventing a good thing if it is forgotten, and re-inventing it again and again. Now in regard to the matter claimed by Dr. Holbrook, I would say that he called on me and told me his price for the right to use his improvement. I promised him this: that I would either pay him his fee of ten dollars or never use it if he explained to me his process. He very frankly told me all about it with that assurance, and I paid the fee to him forthwith, and am fully satisfied with it. I think it an invention that is a very great improvement in many cases. It is intended for lower plates. You all know that in many instances where there are superior plates and mastication is performed only on the lower front teeth, the hard parts pass away and the gums become flabby, which is rather troublesome to dentists, to say nothing about patients. I have used it in two such cases with very satisfactory results. I would not like to dispense with it, and would give ten dollars for any improvement that would give me as much satisfaction as that.

Dr. Buffett.—I would ask if it consists simply in trimming the model where it bears upon the hard parts.

Dr. Berry.—Trimming in that way makes a great deal of difference, but that is not the patented improvement.

Dr. Buffett.—Members of the profession practice that. But it is well to take into consideration the fact that if you trim the model too much, there will be absorption of the tissue under it.

Dr. Berry.—We must, of course, be careful in that respect.

Dr. W. M. Herriott.—Dr. Holbrook is doubtless honest, and thinks he has a very good thing, but it is just, as Dr. Buffett said, the same thing as used by dentists many years since. I used it as long as twenty years ago. By trimming the bearings on the hard parts, the pressure would be taken off of these parts and it would thereby prevent the rocking of the plates, which would occur unless we would use an air chamber or some such principle to remove the pressure on those parts. But this process of Dr. Holbrook's is not new at all, but has been used, as I said, many years by the profession. The point is this: Dr. Berry put himself in a position where he could not help doing that which, as I believe, the doctor did long years ago. I would ask Dr. Berry if that is not true. Didn't you, by the use of the air chamber or trimming your cast, accomplish the same object?

Dr. Berry.—We all know that the wise man Solomon said a long time ago that there is nothing new under the sun. We need not expect anything new, but if anything is forgotten—and I suppose a great many of us have forgotten more than we know now—if anything is forgotten and passed out of use, the man who brings it up again, though he may not have been the originator of it, does a good thing. Now so far as that matter is concerned, I never heard of it before; but I suppose there are forty things I have done as valuable as that, which I may have forgotten. Indeed, I sometimes recollect things I used to do and had forgotten, which prove very valuable to me. This mode of Dr. Holbrook's is not for the fitting of superior plates, but for the lower ones. I have used it with satisfactory results. I put myself in the way of learning something I did not know before.

Dr. F. A. Hunter.—I think we are laboring under a misapprehension in this matter. As I understand it, Dr. Holbrook's invention is not being talked about at all. It is not trimming the model. Dr. Berry has not said what it is. He has paid for it and don't like to say anything about it. It is nothing more than an air chamber.

Dr. H. A. Smith.—Dr. Holbrook's is a patented method. Any, who choose, may refer to the patent records for the mode. Being, all of you, honorable men, of course you would not use it without a license! The method is chiefly applicable to lower sets of teeth, and is simply relieving the bearing upon the ridge, and throwing it mostly upon the edges of the plate. Now if any of you have discovered the secret, it must be owing to your ability to penetrate the mysteries rather than to the clearness of my description of the modus operandi.

Dr. D. R. Jennings.—Dr. George Hurd, of Cleveland, had the same thing patented twelve years ago.

Dr. F. A. Hunter.—Is it a good thing?

Dr. H. A. Smith.—I think it is a good thing if the patient can tolerate a thing of that kind. My idea is that the more bearing a plate has the more comfortably it is worn; that is, if the bearing is equalized both on the hard and soft parts, every portion of the bearing surface doing its work.

Dr. J. H. Warner.—It seems that this new process of mounting artificial teeth is simply an air chamber in lower sets of teeth instead of upper ones. The practice of cutting away any portion of the cast is equivalent to producing an air chamber. It produces undue bearing somewhere, and to that extent is an air chamber. It is an air chamber *in the degree of its pressure.* If cut away to a large extent it is a deep air chamber. If cut away slightly it is still an air chamber. Now, the experience of the profession for twenty or thirty years, has shown to every candid observing man, that an air chamber is, under almost every circumstance, a nuisance. They are something to be avoided whenever and wherever they can be, and whenever an air chamber is produced a vacuum is produced, which fills up sooner or later. An air chamber is of use only for the first few weeks; at least plates worn in this way cause the soft tissues of the mouth to be torn and a state of congestion sets in to the same degree that you reduce the equal bearing of the plate, and to the same extent that you introduce your air chamber. Those who have observed the effect of air chambers, especially those that were made large and deep, ten or fifteen years ago, will agree that air chambers are to be avoided whenever it is possible to do so. It may be proper in very rare cases thus to put in an air chamber and to endure it in the mouth, still it will be of little or no use after it has been worn a month. In my own practice I do not think I have had to use one in twenty years. In a lower plate it is essentially necessary to have all the bearing surface of the plate there can be. Sometimes in taking an impression it will produce an undue pressure on some portion of the mouth, and that undue pressure would be more likely to come upon the superior border of the ridge than upon either side of it, and in some cases it may be necessary to relieve the plate there, and to bring it more on the sides so as to afford relief, but it should at all times be equal.

Dr. J. Taft.—Another plan for the insertion of teeth on artificial plates may be referred to here. I don't know that they are used much in this country, but they are in England. In that style of work the ordinary plates are used, with Ash's teeth put on pivots or standards soldered to the plates, and attaching them with little nuts or sulphur, bringing them down to their place.

I saw, not long ago, a set of teeth of this construction that looked very well, and presented the natural form of the teeth to the tongue in the mouth. Would not this style of work be very applicable for some cases? It is a very easy style of work to construct, the material used being platina, gold, or whatever may be desired. Teeth when broken off, can readily be supplied. In regard to preserving the natural form of the mouth and parts, and as to articulation, it seems at least as good as any method we have. You know, on gold plates, according to the old method, articulation is very frequently injured because of the change of the form of the parts about the tongue. In plates, with this arrangement, that can be restored.

Mr. Ash makes these teeth with tubes running through the crown to the base of the tooth, and they can be very easily regulated and arranged so as to give any form or expression that may be desired. It would be something new in this country, I think.

Dr. Butler.—I would in the first place tike to refer to this method spoken of by Prof. Taft, which is quite commonly used in England, and by the best operators there, in attaching porcelain teeth to plates. The ordinary way is to prepare the plate in the common form of gold or platinum, then grind the teeth with care to secure a close adjustment to the plate. The style of teeth used are known in the market as Claudius Ash & Son's make, of London, with a platinum cylinder through the axis of the tooth. After the teeth have been arranged, a mark is made upon the plate through the cylinder, for soldering the dowels into the plate; then the teeth are slipped on and secured in position with sulphur melted around the pin, and if they want to make a real nice thing, it is made with a little receiving socket along the outer border of the plate, giving a strong bearing for the teeth and .strength of edge to the plate. A pi ce nicely constructed with these teeth, gives a nice presenting surface to the tongue; and then they being set up singly, you can give expression to a set which cannot be obtained with block teeth.

Now, with reference to attaching of crowns to roots of teeth, that has been called up here by reason of claims set up by a man named Richmond, a certain systematic course of manipulations was insisted upon in order to secure practical results, but the old iron bedstead rule is not *always* the most comfortable, to say the least. What we are most interested in is modes that will enable us to manage the various cases in the best manner; no one plan of attachment will secure this end, and the degree of success of any mode recommended will depend upon the skill and care of whoever attempts its use. I have not tried any by this plan upon the incisors, either superior or inferior; but have set them on the cuspids and bicuspids. The plan we would speak of is one that may be pursued in cases where the pulp is alive in the root, the crown being broken down by decay or otherwise. First obtain some measurement of the teeth that will guide you in the construction of the crown for length, then take an impression of the stump and adjoining teeth; having secured a model, you can with care construct a crown ready for setting. Take gold plate twenty carets fine, of sufficient thickness for strength, make a band that will fit tightly around the stump and of sufficient width for length of crown; now solder into one end a piece of gold about one eighth of an inch in thickness, of which to form the cusps; you can now finish it up to correspond in shape to the other teeth; in the final adjustment the stump may have to be trimmed to secure a parallel bearing as nearly as possible where the crown is driven, or, if it be possible to slip a bit of *thin* dam over the stump while working, all the better, carrying it well up under the gum.

Now take your crown and bevel the outer edge of the portion to be telescoped on to the root; having a small hole drilled in the masticating surface to allow any excess of the silicious cement to escape when you drive the crown to its place. The edge of the gold should go fairly up under the margin of the gum. I have set some crowns without the escape, but it requires more care to gauge the amount of cement to fill the interspace. I have attached some crowns in this manner and they have been and are doing good service. I like the Weston insoluble cement, mixed pretty thin, so it gives time to drive the crown home.

I think crowns put on in this way secures the preservation of the root with a living pulp, and less tax on the time and comfort of the patient and operator than is required to make a restoration operation in the ordinary manner, and we may confidently expect them to last as long or longer than the old style of pivoted crowns.

Dr. Corydon Palmer.—In reference to setting gold caps over the stumps as Dr. Butler described, I have something in my own mouth which I prepared entirely myself, performing the whole operation from beginning to end. I pursued the same course described, except that I made a little hole in the top, so that in forcing it down to position the cement might be forced out. It has been worn a considerable time, having been in constant use something over three years. It was done before Richmond became the inventor of his system, or before it was heard of. The inferio bicuspid was made to fit nicely. I put on the dam myself and had already put some cement in and forced it down into its place, being the only fastening which I think is applicable in those cases where there is enough crown to give sufficient support and attachment. If you begin down to the gum or nearly so, as Dr. Horton speaks of, I cannot see how the attachment can be sufficient to hold it.

I would like to say something in reference to the other subject—that of trimming models and fitting plates—because I think all of us have experimented in that way from our first beginning of fitting plates to the mouth. It was one of the first ideas I had advanced to me, or thought of, to trim off a portion of the model here and there, so as to take the bearing off the hard part and put more on the soft parts, equalizing it. It is in the under plates where the most difficulty occurs. In the first place, when taking the impression, the soft part is liable to be forced down too far, so as to give a sharp ridge on the cast, and when we fit it the pressure comes too severely upon the soft tissue, and the patient complains that he cannot wear the plate. Now cutting out the impression, or adding on to the model there, so as to make it deeper along the edge of the ridge would have the same tendency, because it makes the plate to rest upon the soft tissues. I have always found there was where the greatest difficulty occurred, and I have endeavored to take my impressions with the plaster as soft as I could use it so as to prevent the pressing of the soft tissue down too much.

One of the most successful cases I ever had was where the ridge was quite prominent. I so formed the plate that it did not touch the ridge at all. When put into the mouth it did not seem to fit very well, but it gradually settled down to its place and finally set very well, and I never had to make any alterations in it though put in before the war. So I think there is more necessity of relieving the middle of the plate than of adding on to the model, so as to make them press harder.

Dr. C. Von Bonhorst.—With regard to fitting lower plates I have had some little experience, and that experience has proven that it makes but little difference as to what the plates are made of, providing that the adaptation is perfect, and that the impression is correctly taken, and that the edges of the plate do not impinge too much upon the soft tissues; and where there is an irregularity or a tendency to cause irritation by too sharp an edge, either on the outside or inside of the ridge, I have invariably made it a rule to round off these edges If made of gold plate, I solder around the edge of the plate a half round gold wire, enabling me to give a round edge where it comes in contact with the soft tissues. Where the plate is made of rubber, this is done more easily by making the edges a little thicker at the start. I always avoid getting undue pressure upon the soft tissues, but have it come where it properly belongs, viz: on the hard portion of the ridge. Many abject failures are caused by the belief that the plate must run down as deep as possible in order to get any suction, and get the plate to stay there. But a shallow, well fitting plate will give much more satisfaction to the wearer than a deeper one that infringes upon the soft tissues and movable muscles, as the motion in eating or talking will throw the plate off.

In regard to the suction in upper plates, I rarely use an air chamber. After making the cast I study the muscular attachments, then with a half round, pointed instrument mark a line all around where I intend the edge of the plate to terminate when finished. This makes an indentation in the cast; of course a corresponding elevation in the plate, when of rubber, near the edge. "In the case of gold plate solder on a half round piece of wire all around the plate, as first stated." The marking out on the cast also serves as a guide in filing up the plate, so as to save trouble in doing so after the patient comes to have it fitted, as you have made your calculations before. This little ridge formed by the marking on the cast, or the soldering on of the wire, makes a slight indentation on the soft tissue, and consequently obviates the necessity of an air chamber, and where it impinges a little too much, is easily relieved by filing down at the point indicated.

You thus equalize the pressure all over the roof of the mouth, and avoid too much pressure on the palatal artery, which is a matter entirely ignored by many dentists. The plate may not adhere at first like one with an air chamber, but explain to the patient the difference, and that after a few days or weeks wearing, all will be right. They will then not expect too much at the start, but will give you credit for honesty, which is a factor of no small importance in the reputation of a dentist.

[TO BE CONTINUED.]

